# A Mobile Health Platform for Self-Management of Pediatric Cystic Fibrosis: Qualitative Study of Adaptation to Stakeholder Needs and Integration in Clinical Settings

**DOI:** 10.2196/19413

**Published:** 2021-01-26

**Authors:** Sarah B Rutland, Rikard Palmer Bergquist, Andreas Hager, Robin Geurs, Cathy Mims, Hector H Gutierrez, Gabriela R Oates

**Affiliations:** 1 Pediatric Pulmonary and Sleep Medicine The University of Alabama at Birmingham Birmingham, AL United States; 2 Motivo Management LLC Reno, NV United States; 3 Upstream Dream AB Stockholm Sweden; 4 Children's of Alabama Birmingham, AL United States

**Keywords:** cystic fibrosis, mHealth

## Abstract

**Background:**

Cystic fibrosis (CF) is an inherited chronic condition that requires extensive daily care and quarterly clinic visits with a multidisciplinary care team. The limited exchange of information outside of the quarterly clinic visits impedes optimal disease self-management, patient engagement, and shared decision making.

**Objective:**

The aim of this study is to adapt a mobile health (mHealth) app originally developed in Sweden to the needs of patients, families, and health care providers in a CF center in the United States and to test it as a platform for sharing patient-generated health data with the CF health care team.

**Methods:**

Focus groups with health care providers of patients with CF, adolescents with CF, and caregivers of children with CF were conducted to determine what modifications were necessary. Focus group data were analyzed using a thematic analysis, and emergent themes were ranked according to desirability and technical feasibility. The mHealth platform was then modified to meet the identified needs and preferences, and the flow of patient-generated health data to a secure Research Electronic Data Capture database was tested. Protocols for data management and clinical follow-up were also developed.

**Results:**

A total of 5 focus groups with 21 participants were conducted. Recommended modifications pertained to all functionalities of the mHealth platform, including tracking of symptoms, treatments, and activities of daily care; creating and organizing medication lists and setting up reminders; generating reports for the health care team; language and presentation; sharing and privacy; and settings and accounts. Overall, health care providers recommended changes to align the mHealth platform with US standards of care, people with CF and their caregivers requested more tracking functionalities, and both groups suggested the inclusion of a mental health tracker as well as more detailed response options and precise language. Beta testers of the modified platform reported issues related to translatability to US environment and various bugs.

**Conclusions:**

This study demonstrated the importance of identifying the needs and preferences of target users and stakeholders before adopting existing mHealth solutions. All relevant perspectives, including those of clinicians, patients, and caregivers, should be thoroughly considered to meet both end users’ needs and evidence-based practice recommendations.

## Introduction

### Background

Cystic fibrosis (CF) is the second most common inherited disorder in the United States. The disease affects multiple systems, including the respiratory, digestive, endocrine, and reproductive systems, and involves a complex, time-consuming daily care routine with multiple oral and inhaled medications, airway clearance therapy, diet, and exercise [1,2]. In the United States, clinical care guidelines recommend quarterly multidisciplinary CF clinic visits. During such visits, patients and families are asked to remember and communicate to the clinical team the most relevant aspects of their disease experience from the previous 3 months. From that snapshot, clinicians are expected to gather enough detail to make optimal treatment recommendations [3]. Although the majority of care takes place at home, exchange of information between the patient and the clinical team outside of the clinical setting is currently limited and occurs mostly via phone calls. This traditional, episodic model of care delivery is not well suited for a chronic condition such as CF and does not support optimal disease self-management, patient engagement, and shared decision making [3-6]. For optimal CF care, a bidirectional patient-clinician communication that takes place between visits is necessary. Such communication is particularly important for children and adolescents who are learning to transition from clinician-regulated care to autonomous self-management of their disease [7,8]. Ideally, the bidirectional communication would occur with electronic tracking of patient-reported symptoms, real-time sharing of this patient-generated health data controlled by the patient but matching the requirements of clinical workflows, and timely clinical feedback to such data [3,9].

Research in other complex chronic conditions has shown that patients benefit from routine collection of patient-generated health data. Studies have reported associations between the use of patient-generated health data and improved symptom control and quality of life [10-14], patient-clinician communication and satisfaction [15,16], reduced health care utilization, and increased survival [17-20]. Such an approach is not yet used in CF clinical practice, despite a strongly stated need by the CF patient community [21-23]. A previous assessment of preferences for remote collection and sharing of patient-generated health data among patients with CF or their caregivers reported that the majority are willing to share such data [24].

The opportunities for collaborative, continuous, and data-driven chronic care have increased exponentially with the application of mobile health (mHealth) technologies. Mobile apps allow patients to collect, monitor, and report symptoms and events outside of an office visit and to make these data available to providers for shared decision making. mHealth interventions have been widely used in the management of diabetes, hypertension, and chronic pain, among others [13,14,20]. Although technology has been used to facilitate earlier detection of pulmonary exacerbations and adherence monitoring in CF care [25-27], mHealth solutions for CF self-management have not been adopted [28,29]. CF care is uniquely suitable for mHealth solutions because of well-established clinical guidelines, multidisciplinary care teams, national patient registry, and an expansive network of accredited CF centers; however, barriers related to the integration of patient-generated health data with information technology (IT) systems and electronic medical records have been documented [21]. In addition, the few existing CF apps only partially satisfy the user needs reported in previous research [29,30]. mHealth solutions are even more important in the context of the COVID-19 pandemic, which has imposed limitations on in-person visits for chronic care. As such, mHealth approaches may be used to supplement telehealth as a risk-reduction strategy [31].

### Genia

**Figure 1:**
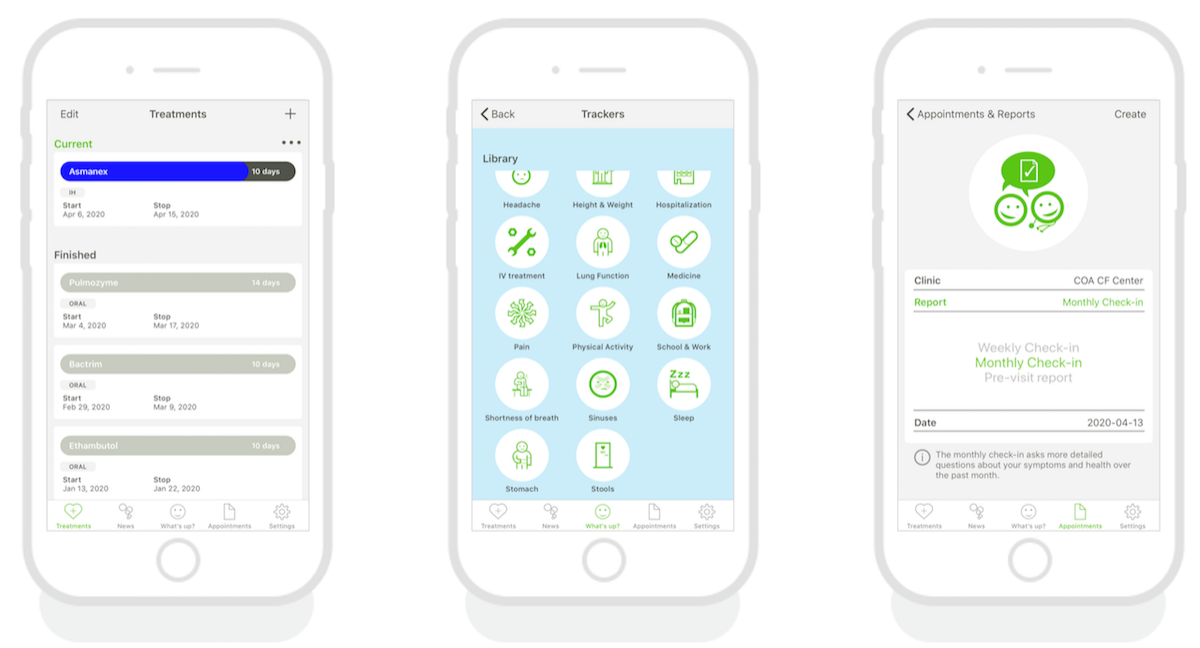
Genia dashboards: (A) treatments, (B) trackers, and (C) appointments and reports.

Genia is a mHealth platform designed to facilitate the coproduction of CF care (Figure 1). Developed by Upstream Dream in collaboration with the Swedish CF community at Karolinska Institute in 2014, it was first introduced to pediatric CF centers in Sweden and has been well received by patients, with 65% to 87% of families using the app at the most active clinics to track symptoms, activities, and aspects of daily care and share them with the clinical team [32].

Genia supports the upload of images (eg, a patient may take a photo of their sputum and upload it via the app) and can connect to other apps or devices, such as electronic spirometers and smart medication dispensers. A proof-of-concept test of the Genia platform in juvenile idiopathic arthritis reported that it improves patient engagement, patient-centered care, and practice-based learning and recommended the uptake of the platform in other chronic conditions [33,34].

### Objectives

This study adapted Genia to the needs and preferences of the CF community in a pediatric CF center in the United States. A proof-of-concept testing was conducted to optimize the platform, remove glitches, and ensure the security and privacy of patient-generated health data.

## Methods

### Theoretical Model

The project is informed by the model of health care service coproduction [5] whose central tenet is that health care services are coproduced by patients and providers in systems that support and constrain effective partnerships.

### Study Design

#### Determining the Needs and Preferences of the CF Community

We used focus groups to assess the needs and preferences of the pediatric patients with CF, their families, and their health care providers regarding the Genia mHealth platform and to determine what modifications were necessary. Patient focus groups were conducted via videoconferencing in keeping with infection control guidelines [35]. For accessibility and convenience, caregiver focus groups were conducted in 3 different locations in the catchment area of the University of Alabama at Birmingham/Children’s of Alabama (UAB/COA) CF center. UAB/COA is the only pediatric CF program in Alabama, serving families from across the state. Patients and familial caregivers were English-speaking and were recruited during routine clinic visits and by phone. Participants provided informed consent and received a US $25 gift card to compensate for their time. The clinical focus group was conducted on site at the UAB/COA CF center during a routine meeting of the CF clinical team. As such, clinical participants were not compensated for their time.

During caregiver focus groups, participants were first shown a 2-minute video that demonstrated the functionalities of the existing Genia app and then asked to explore the app on their own using study iPads preloaded with a beta version of the app in English. Moderators were available to answer questions about the app. Patients who participated in a virtual focus group were given access to the app on their personal devices up to 3 days before the focus group. As they had the opportunity to explore the app beforehand, less time was devoted to app demonstration. Focus group sessions were led by 3 research team members (GO, SR, and RG) who used a semistructured guide (Table 1) to facilitate discussions. Each session lasted approximately 90 minutes. Focus groups were recorded, transcribed verbatim, and deidentified for analysis. The study was approved by the Institutional Review Board of the University of Alabama at Birmingham (Protocol IRB-300001749).

**Table 1:** Semistructured guide for focus group discussions.

Question type	Questions
General	What are your first impressions of the app? How do you see yourself using the app? What part is most interesting to you? What part is most useful to you? What do you think about the visual presentation? What could be improved in the app?
Overarching	How should the Genia platform be adapted to meet your needs and preferences?
Feature specific	Trackers: Are these the topics you would track and talk to your doctor about? Are there other important topics that are not included? Previsit reports: What do you think about the questions? Is there something that is not clear or could be improved? Treatments: What do you think about this feature? Is there anything that could be improved? Appointments: What do you think about this feature? Is there anything that could be improved? News: What do you think about this feature? Is there anything that could be improved? Settings: Is there anything that could be improved in the app settings?

Data from all focus groups were combined and analyzed together using a thematic analysis [36], an interpretative research approach that uses a purely qualitative account of data rather than frequency of codes for theme development [37]. Thematic analysis identifies themes from both latent and manifest content, without considering code frequency. Thus, the number of focus groups, participants, or categories had no consequence on the results. A constant comparative method [38] was employed to generate categories and themes. Transcriptions were coded independently by 3 research team members (GO, RR, and RG), and then, codes were discussed by the entire research team and the final coding scheme was decided jointly. The Genia app developers did not participate in the data analysis and were blinded to the identity of the participants.

#### Adapting the Genia App to Identified Needs and Preferences

Focus group data were first categorized according to app features. The research team then ranked participant recommendations in 3 categories (highly desired, moderately desired, and less desired) based on participant feedback and sent the recommendations to the Genia developers. The developers categorized these recommendations according to technical feasibility (immediately feasible, feasible in the future, and not feasible) based on app infrastructure, time required to make the changes, and cost. The research team and Genia developers met to discuss what recommendations were both highly or moderately desired and immediately feasible. The mHealth platform was then modified according to these recommendations and prepared for the proof-of-concept testing phase.

#### Testing the Modified Platform

In this phase, we tested the Health Insurance Portability and Accountability Act (HIPAA)–compliant data flow from the app to a secure study server housed at UAB/COA and beta tested the modified platform. To ensure the security and privacy of patient-generated health data, we tested the transmission of patient-generated reports sent as HIPAA-compliant PDF and .xls files to a secure Research Electronic Data Capture (REDCap) server at our academic institution. REDCap, which uses cellular networks and encrypted point-to-point communication, is a secure web app specifically designed to support and house web-based data capture for research studies. We enrolled 6 participants from the caregiver focus groups to test data flow to the server and to report bugs and other problems in the mobile app for 30 days. Participants provided informed consent and were compensated US $100 for their time. Additional beta testing of the modified app was performed by research team members (GO, RR, RG, and CM).

#### Development of Data Management and Clinical Follow-Up Protocols

In this phase, we developed protocols for the management of patient-generated health data submitted to REDCap and for clinical follow-up in response to submitted data. Clinical protocols were developed with input from the COA CF Nurse Coordinator and the UAB/COA CF Center Director. We also trained the CF care team on the use of the platform in a one-day face-to-face session.

## Results

### Determining the Needs and Preferences of the CF Community

A total of 5 focus groups with 21 participants were conducted: 1 with pediatric clinical team members (n=5), 1 with adolescent patients with CF treated at COA (n=5), and 3 with familial caregivers of pediatric patients with CF (n=11). Clinical team members represented the specialties included in a multidisciplinary CF clinic (pulmonology, nursing, nutrition, respiratory therapy, and social work). Patients with CF, mean age 17.8 years (SD 1.7; range 15-20 years) and 80% (4/5) female, resided in 4 different geographic areas of the state. Familial caregivers, mean age 42 years (SD 6.9; range 33-59 years), 90% (10/11) female, and 90% (10/11) parents, represented 3 different geographic areas of residence.

#### Trackers

The Genia app allows users to keep track of their symptoms using a library of developed trackers. Clinical, patient, and caregiver respondents prompted developers to create or improve trackers for a variety of health measures and physical symptoms. Overall, all groups requested that existing trackers be more specific. For example, they recommended that the pain tracker be modified to ask about the location of the pain, its severity, and how long it has been occurring. In addition, all groups recommended a tracker for stool composition and frequency. Caregivers shared that trackers for seemingly embarrassing topics such as stools could help older children better communicate their symptoms:

…because when they get to the age of 17 and 13, they don’t wanna discuss it at all. That is gross, and they don’t wanna talk about it...When my children were younger, I changed their diapers. I went in the bathroom with them, and you could check. That don’t happen any longer, so to have an app that [...] they can put in there when they see, and then it’s not necessarily “I’ve got to go tell mom this is what it looked like."

Caregiver

Overall, patients and caregivers requested more detailed clinical trackers, whereas the clinical team considered such trackers unnecessary. For example, contrary to the opinion of the clinical team, patients and caregivers wanted to have a lung function tracker to input the latest pulmonary function test results from each clinic visit and track changes over time, a hospitalization tracker to keep record of when and for how long they are hospitalized, and a vaccinations tracker to help keep track of flu shots and other vaccinations. They also requested a tracker for sinus symptoms, shortness of breath and oxygen saturation, and CF-related diabetes. In addition, caregivers requested that the separate height and weight trackers be combined into one for convenience and ease of use and that the nighttime cough and daytime cough trackers be combined as well. Patients recommended that the exercise tracker be renamed to *physical activity*, with a drop-down menu of options, including various sports, yoga, dancing, hiking, or playing outside, among others.

When asked what trackers were missing from the current list, all respondents highlighted the importance of tracking mental health:

Yeah, a mental health question would be very, very helpful ... for the parents and child, as well, because it gives them a better insight into what’s going on...

Caregiver

I would like to see if we could add in mental health [...] anxiety or stuff like that because we— that’s something that some of us get asked at clinic and stuff, and sometimes we don’t remember it, so that would be a really good one to add to it.

Patient

Caregivers hoped that children who are not comfortable talking to their parents about their mental health would tell the app how they are feeling and share these data with the CF care team:

I'm okay with leaving it and let him feel comfortable. I'm not gonna try to look and see what he's writing.

Caregiver

Clinical participants recommended including a mental health tracker with questions about depression and anxiety from the 4-item Patient Health Questionnaire [39]. In addition, patients and caregivers recommended creating an emotions tracker, where users can choose from a drop-down list of emotions and elaborate on why they feel that way:

Call the button daily emotions, because you want them to put it in every day so we can keep track of it, so I think that’s under emotions.

Caregiver

The one I’m thinking is just your mood [...] Were you happy today? Was there anything that you made feel sad? Something like that.

Patient

#### Creating a Treatment

The Genia app allows patients to track medications and treatments, including frequency and duration, and to set treatment reminders. Clinical participants requested updating the treatment and medication list to the US context, to match current CF clinical care guidelines. Caregivers requested including a medication dosage in addition to the medication name and an option to print their medication list from the app for sharing with others, such as pediatricians, school nurses, or daycare providers. They wanted to be able to set multiple daily reminders for the same treatment and reminders for treatments that cycle on and off (eg, antibiotics taken on 28 days on/28 days off schedule). Caregivers also asked for the ability to enter the prescription date and to set refill reminders for medications, medical supplies (eg, nebulizer cups), or treatment-related tasks (eg, changing air filters and flushing a port). Finally, caregivers wanted the flexibility to reorder their medication lists as necessary for different purposes, for example, alphabetically, by time of treatment (morning vs evening), by type of treatment (inhaled vs oral), or by organ system (digestive vs respiratory).

#### Exporting Reports to the Clinical Setting

One of the most important features of the Genia patient support system is sending patient-generated health data to the care team in the form of reports in a PDF format before clinic appointments. Caregivers and patients wanted to make sure that there was a place to keep a list of questions for an upcoming visit. Caregivers also strongly suggested appending the previsit reports to also include the pen-and-paper questionnaire traditionally administered in the clinic waiting room:

[...] ’cause I’ve been to the point where I’m like I’m just not gonna fill it out.

Caregiver

I find the papers extremely stressful. [...] I’m trying to have [my kids] not kill each other, and I’m trying to fill out— then the people keep coming in, and I’m trying to answer questions. I just get stressed out about trying to fill out the papers.

Caregiver

Caregivers particularly appreciated the opportunity to take notes during the clinic visit and keep them in the app as a personal record of what was discussed or what changes were made during that visit, along with vital signs, laboratory test results, and results from pulmonary function testing.

#### Language and Communication

Both caregivers and clinicians suggested multiple changes to the current app language. Some of these changes reflected differences in terminology between the Swedish and US context (eg, breathing treatments vs airway clearance), whereas others pertained to the use of a more idiomatic English language. Several caregivers expressed that there should always be a free-text option or a comment box to encourage patients to share details about their observations and encourage open communication. Caregivers of adolescents were particularly worried that their children would not communicate enough information in the app without detailed prompts.

#### Sharing and Privacy

Respondents had different opinions about sharing and privacy within the app by role (patient vs caregiver) and age. Adolescent patients thought that their caregivers did not need to be heavily involved in their app usage:

I’m 15, and I think it’s a great way for growing up—a great way to learn out on your own. [...] I would learn when to take my medications and to refill them and different things like that.

However, some caregivers had mixed feelings: although they wanted to give their children privacy, they also wanted to be aware of what their children were entering in the app:

[...] still, I hate the fact that there’s things I just don’t know.

Caregiver

Other caregivers believed that children should not be able to keep anything private in the app if they are not legal-age adults:

[...] as a parent, my child can’t keep anything from me until they turn 18.

Caregivers recommended making all notes private by default to encourage people to record their true thoughts in the app. Users can then choose what notes to share with the CF care team when they generate their previsit report.

#### Settings and Account

These features were consistently important points of discussion for caregivers and patients. Caregivers of multiple children with CF wanted to be able to track their different children seamlessly, without having to log off from one profile and log on to another:

Eventually if they could pick out their color scheme or they could add their own picture. That way at a glance I know if I’m looking at [Son’s] or I’m lookin’ at [Daughter’s], and I don’t have to go back and be like, whose is this?

Caregivers of younger patients wanted both the parent and the child to be able to track and view symptoms and treatments. Both caregivers and patients requested the ability to personalize their settings with profile pictures, avatars, and color schemes.

#### News

Caregivers suggested that the news feature of the app should be used to maintain current information about the CF care team, including their names, photos, role in the team, and contact information. Caregivers also wanted to receive frequent updates about clinical trials, research news, medication discounts, and CF educational events and resources:

I don’t think you can ever get too much information. As parents, we can never get too much information.

### Adapting the Genia App to Identified Needs and Preferences

The results of the focus groups highlighted both overlapping and differing preferences among clinical staff, caregivers, and patients. Overall, when making decisions about modifications, the research and development teams prioritized the perspectives of patients and caregivers—the app’s end users—over those of clinical team members, which resulted in a greater number of trackers. Although not every recommendation made by participants was feasible, most suggestions were successfully incorporated into the modified app, highlighting the necessity of identifying the needs and preferences of the end users before entering a test environment. A summary of the modifications made as a result of focus group recommendations is presented in Table 2. A full list of all changes is provided in Multimedia Appendix 1.

**Table 2:** Summary of modifications.

Type	Extent		
	New	Revision	Planned for 2.0 release
Trackers	Emotions Hospitalizations Intravenous treatments Mental health Shortness of breath Sinuses Stomach Stools	Airway clearance Cough Energy Headache Height and weight Lung function Medicine Pain Physical activity School and work Sleep	CF^a^-related diabetes Diagnosis and mutations Goals, with reminders and rewards Vaccinations
Treatments	“Other” option Printing list of medications Reorganizing medications	US-specific medications and treatments Multiple reminder options	Equipment reminders Medication dosage Refill reminders
Reports	Adding clinical history form Notes for upcoming visit Notes during visit Weekly and monthly check-in	N/A^b^	N/A
Language and presentation	N/A	Personalization with a user name Idiomatic English	Avatars and color options
Privacy	N/A	Default “do not share”	N/A
Settings	Sync other health apps	Parent and child track under the same account Technical support contact	Calendar Clinical team contact information Within-app messages to the clinical team

^a^CF: cystic fibrosis.

^b^N/A: not applicable.

### Testing the Modified Platform for Technical Issues

Testing of the HIPAA-compliant data flow from the patient-facing app to a secure US-based cloud server to the REDCap study database was necessary to ensure the protection and privacy of patient-generated health data. The research and development teams also tested that patient-generated reports in the mobile app were correctly uploaded to the REDCap database. The issue of data readability for the clinical team and the research team had to be addressed as well. The development team successfully converted the incoming patient-generated reports into PDF files with charts, graphs, and text boxes of tracked symptoms and raw data in .xls files. This ensured that the clinical team had an easy way to interpret the output of patient symptoms and that the research team had easily accessible data for data analysis.

Beta testing of the modified Genia platform for technical issues was conducted for 30 days with 6 participants (mean age 41 years, SD 2.4; range 43-51) from the pool of caregiver focus group participants, as they were already familiar with the platform. Specifically, we asked participants to test every tracker, submit notes with and without pictures, test if the treatment reminders feature is functional, and make note of anything that needs to be corrected. The research team informed testers that data entered into the app would not be acted upon by the clinical team during the beta testing period and that they should contact the clinical team through usual channels if health concerns arose.

Beta testers reported bugs and issues to a contact person on the research team who then funneled that information to the development team. The most common issues reported by beta testers included metric units for height and weight, unnecessary caps on information input, medication reminders not going off, and leftover Swedish language.

The developers removed the reported glitches and worked collaboratively with the research and clinical team members to make adaptations that ensure smooth user experience. For example, beta testers reported a medication missing from the menu of options. The CF nurse coordinator reviewed and updated the medication list, and the developers updated the app accordingly. In addition, beta testers reported an issue with attaching images to previsit reports, and the research team detected a glitch in the upload of previsit reports to the REDCap database. All these issues were fixed by app developers. A complete list of optimizations and removed glitches is provided in Multimedia Appendix 1.

### Development of Data Management and Clinical Follow-Up Protocols

The clinical and research teams met with the development team during a site visit in November 2019. This meeting was instrumental in laying the groundwork of clinical protocols. For example, clinical providers had concerns about how the patient-generated health data from the app would fit into clinical planning and flow. To address these concerns, a clinical management protocol was developed that summarizes patient reports from the app and incorporates them into the weekly previsit overview of patients with an upcoming clinical appointment that week. To ease strain on the providers, an appointed research team member organizes the patient reports from REDCap and provides the PDFs to the CF nurse coordinator on a weekly basis. The CF nurse coordinator then follows a patient support protocol that directs what needs to be done with specific types of information (eg, >3 increased symptoms for >2 days→report to treating pulmonologist and add antibiotic and increased depression reports→initiate contact with a social worker or mental health counselor).

The effect of the platform for disease self-management, patient-reported outcomes, and patient-centered care is being assessed in an ongoing clinical trial (NCT03910881).

## Discussion

### Principal Findings

In this study, we adapted an mHealth platform developed for the CF community in Sweden to the needs and preferences of the US CF community. We also conducted proof-of-concept testing to optimize the platform, remove glitches, and ensure the security and privacy of patient-generated health data. The project demonstrated the importance of identifying the needs and preferences of end users before using existing mHealth solutions and the need to consider all relevant perspectives, including those of clinicians, patients, caregivers, and app developers.

Although end user preferences guided the modification of app functionalities and presentation, clinician perspectives governed the development of clinical protocols that detailed how patient-generated health data are used in clinical settings. Successful coproduction of health care services for managing a chronic health condition [5] requires the perspectives of both parties. Engaging end users in the development or modification of mHealth apps can help prevent implementation challenges and increase the likelihood of app uptake by the target audience [40]. Engaging clinicians can ensure that mHealth solutions are practical in clinical settings and can be integrated into the clinical data flow within existing IT environments [21]. Other barriers and challenges also exist. For example, not all end users may be able or willing to self-monitor and report symptoms or activities, particularly those with fewer resources or lower literacy, whereas clinicians may find the use of patient-generated health data burdensome or having little measurable effect on health outcomes. Addressing the priorities and concerns of both parties will facilitate realistic and attainable goals in the context of coproduction facilitated by technology-enabled data sharing [5]. For successful mHealth interventions, patient-generated health data must be both meaningful to patients and useful to clinicians [22].

The preferences of end users of Genia mirrored the preferences of patients with CF and their caregivers reported in a previous study [30]. Specifically, end users preferred an app with multiple functions that facilitates access to information, automates disease management, integrates with other apps, facilitates communication with the health care team, and is highly customizable to meet individual goals and preferences. Involving end users in all stages of mHealth development and collaborating with clinicians and health care system experts may result in apps that maintain engagement, improve coproduction of services, and ultimately impact self-management and health outcomes [30].

The described modification process represents a scalable approach to adapting mHealth solutions to local needs and changing contexts. Feedback from patients, caregivers, health care providers, and experts gave input to developers on how to prepare the mobile platform for further adaptations to local needs and changing contexts. For example, a file-based approach was developed to support a multilanguage platform, and medication and treatment lists were prepared to allow for dynamic updates and changes in response to clinician requests and patient preferences. The collection of patient-generated health data was implemented according to a structure that could be used to create new trackers and new report questions. The result was a more scalable and dynamic platform, facilitating further adaptations. Such an approach can be replicated in further modification of this and other mHealth platforms to track home spirometry, home intravenous antibiotics, oral and nebulized medications, or airway clearance therapy via smart devices.

App repositories include hundreds of apps that claim to improve disease management, health outcomes, and health-related behaviors. However, few are evidence-based solutions developed with the involvement of health care professionals, patients, caregivers, and behavioral scientists [33,34]. Even fewer are tailored to the components of daily CF care and the symptoms typically experienced by people with CF. The few apps that collect CF-specific patient-generated health data are not integrated in the CF care plan or the clinical workflow. Without such integration, mHealth technologies can have only a minimal impact on chronic disease care and management [41]. At the same time, integration of patient-generated health data in clinical care via mHealth solutions needs to be done in a way that safeguards the privacy and integrity of patients in their domestic domain [42]. mHealth platforms and patient support systems, such as Genia, provide new opportunities for improved self-care and clinical management of CF. However, for the integration of mHealth solutions in clinical care, their feasibility, their acceptability to patients and providers, and their clinical effectiveness need to be tested in rigorous clinical trials.

### Limitations

Participants included English-speaking patients, caregivers, and health care providers recruited from a single pediatric CF center in the United States. The perspectives of this convenience sample may not be representative of all CF centers or geographic areas in the country. However, as CF disease management and care delivery are highly protocolized across the network of accredited CF centers, vast differences between CF stakeholders from other centers and regions are unlikely. In addition, focus group participants had an opportunity to use the app only during focus group discussions, which may have limited the scope and depth of their experience and feedback. Finally, the project did not address data mapping of patient-generated health data to interoperable standards to integrate Genia-collected patient-generated health data in the electronic health record, a step that will be addressed in a future study phase.

### Future Directions

A currently ongoing pilot clinical trial (NCT03910881) assesses whether the use of Genia over 6 months enhances patient-centered care (shared decision making, patient activation, patient satisfaction, and patient self-efficacy) and improves patient-reported outcomes (symptom scales and quality-of-life domains of the Cystic Fibrosis Questionnaire-Revised [43]). A randomized clinical trial will evaluate the impact of Genia on respiratory and nutritional outcomes. Informed by the model of health care service coproduction [5], these study phases are predicated on the hypotheses that (1) use of patient-generated health data will affect providers’ actions, which will have downstream benefits, and (2) use of patient-generated health data will affect patients’ disease management behaviors and interaction with the health system.

### Conclusions

mHealth offers new opportunities to support self-management of CF, facilitate patient-physician communication, and promote coproduction of care. Broad and successful uptake of mHealth solutions in CF care requires careful consideration of patient and family perspectives and active involvement of CF clinical teams.

## Appendix

Multimedia Appendix 1 Detailed description of changes based on focus groups and beta testing: implemented, not implemented, and planned.

## Abbreviations

CF: cystic fibrosis
COA: Children’s of Alabama
HIPAA: Health Insurance Portability and Accountability Act
mHealth: mobile health
REDCap: Research Electronic Data Capture
UAB: University of Alabama at Birmingham
