# Supplementary material for: A Mobile Health Platform for Self-Management of Pediatric Cystic Fibrosis: Qualitative Study of Adaptation to Stakeholder Needs and Integration in Clinical Settings
Source: JMIR Form Res. 2021 Jan 26;5(1):e19413. doi: 10.2196/19413 (PMC7872830; doi:10.2196/19413)
Supplement: Multimedia Appendix 1 [file formative_v5i1e19413_app1.docx]

| **Supplemental Table 1. Detailed description of changes based on focus groups and beta-testing: implemented, not implemented, and planned** |
| --- |
| **Focus group input: implemented changes** |
| Create two new reports: weekly and monthly check-in |
| Include history form in pre-visit report so users don’t have to fill it out as a paper form in clinic |
| Add a tracker Stools: What is your stool like? (Formed, Loose, Oily, Hard, Bulky) How many times per day? (<1, 1, 2, 3, >3) |
| Add a tracker Mental health: Ask about depression and anxiety |
| Combine Height and Weight trackers into one |
| Rename Breathing exercises tracker to Airway clearance. Ask, “How many times did you do it?” “How long did you do it?” “With huff cough or without?” |
| Change Exercise to Physical activity. Add list of sports, including yoga, martial arts, etc. |
| Keep the Lung function tracker to log in PFTs from clinic |
| Add a tracker for Hospitalizations |
| Add a tracker for Shortness of breath and oxygen saturation |
| Add a function for noting questions in preparation for upcoming visits |
| Add During Meeting Notes for personal record of what was discussed or decided during that visit |
| Personalize “What’s up?” with name of child; address the child by their name |
| Allow for both the child and parent to track under the same account |
| Customize the user profile to let the user upload their own picture or image |
| Add a Find medication function to input common medications and treatments that are not in the standard list |
| Add an option to print the list of medications |
| Allow reminders to go off multiple times a day for medications or treatments taken multiple times daily |
| Notes section: Add a Diary or Journal for making notes |
| Adapt the Diary/Notes functions to have a text area where you can add reminders |
| Default settings for sharing Notes, Observations, and Comments in reports should be “No”; sharing should require active choice. “Yes, I want to share it” |
| Functionality to sync other health apps; e.g. steps, weight, spirometry (via Apple Health) |
| Implement a front-end "router" so the user can select the country of their CF Center (e.g., USA, Sweden) |
| **Focus group input: planned changes** |
| Add dosage to medications |
| Reminders: For medications, have an option to enter a prescription date and set refill reminders |
| Reminders: Add an Equipment option and set refill reminder |
| Add a tracker for CF-related Diabetes to track blood sugar levels, symptoms, etc. |
| Add a tracker for Vaccinations to track flu shots and other vaccinations |
| Add a tracker for Goals to set goals for oneself between appointments; include an option to set push notifications to remind you of your goals. When a goal is reached, add a star, unlock colors, etc. |
| Have rewards (stars, points) for doing treatments so a child can be recognized when in clinic |
| Pre-visit reports: After generating report, notify user if the visit will include labs, x-ray, or other procedures |
| Settings: Be able to choose the trackers that the user wants so there not too many trackers on the homepage |
| A tab with name, photo and contact information for the clinic team—my dietitian, my RT, etc |
| Add a calendar feature that can sync with my school schedule, my exercise schedule, etc. |
| Profile: Include in the profile PFTs, Hospitalizations, CFRD, CFTR mutations, and when you joined the app |
| Have option to send messages to the clinic so that we don’t have to make a phone call |
| **Beta-testing: implemented changes** |
| Grey out (make not selectable) the Date for Weekly and Monthly Check-in. |
| Include text to explain that the Pre-visit Report Date is the Clinic Visit Date |
| Remove mandatory setting for answers to questions in reports |
| Add standard reminders of 3, 7, and 10 days for Pre-visit Reports |
| Add standard weekly (Sundays) reminders for weekly check-in reports |
| Add standard monthly (last day of month) reminders for monthly check-in reports |
| Update and add info text for weekly, monthly, and pre-visit reports, explaining the purpose of each |
| Update the information text in the pre-visit report reminder |
| Edit/Change the date of the clinical visit in an already created pre-visit report |
| Add FEV1% and FCV with liter readings to the lung function tracker |
| Update Settings with contact info (phone number and email for technical support) |
| Add inches and ounces to the combined Height and Weight tracker. |
| Add an option to choose or add all observations when selecting trackers in Reporting |
| Search in the list of treatments and add a found medicine |
| Create a default list of treatments/medications and import updated lists as necessary |
| Allow for larger (>Mb) pictures in the Notes field, attachment |
| Remove the cropping frame when adding picture using the camera or from the photo album |
| Fix the size limit and the scaling of pictures in the News feed |
| Implement a back-office notification when a user report fails to upload to the REDCap server |
| Implement an email notification when a report is uploaded to the REDCap server |
| Update the end-user agreement |
| Update all left-over Swedish text to U.S. English |
